# Feasibility results of an electromagnetic compatibility test protocol to evaluate medical devices to radio frequency identification exposure

**DOI:** 10.1186/1475-925X-13-110

**Published:** 2014-08-03

**Authors:** Seth J Seidman, Omar Bekdash, Joshua Guag, Maryam Mehryar, Paul Booth, Paul Frisch

**Affiliations:** 1 FDA / CDRH / OSEL, 10903 New Hampshire Ave, WO Bldg 62 Room 1134, Silver Spring, MD 20993, USA; 2 FDA / CDRH / OSEL, 10903 New Hampshire Ave, WO Bldg 62 Room G105, Silver Spring, MD 20993, USA; 3 Memorial Sloan-Kettering Cancer Center Department of Medical Physics, 1275 York Ave, New York, NY 10065, USA

**Keywords:** Electromagnetic compatibility, Electromagnetic interference, EMC, EMI, Radio-frequency identification, RFID, Medical device, Test methods, Simulator

## Abstract

**Background:**

The use of radio frequency identification (RFID) systems in healthcare is increasing, and concerns for electromagnetic compatibility (EMC) pose one of the biggest obstacles for widespread adoption. Numerous studies have demonstrated that RFID systems can interfere with medical devices; however, the majority of past studies relied on time-consuming and burdensome test schemes based on ad hoc test methods applied to individual RFID systems.

**Methods:**

This paper presents the results of using an RFID simulator that allows for faster evaluation of RFID-medical device EMC against a library of RFID test signals at various field strengths.

**Results:**

The results of these tests demonstrate the feasibility and adequacy of simulator testing and can be used to support its incorporation into applicable consensus standards.

**Conclusions:**

This work can aid the medical device community in better assessing the risks associated with medical device exposure to RFID.

## Background

The use of radio frequency identification (RFID) systems in healthcare is increasing and concerns for electromagnetic compatibility (EMC) pose one of the biggest obstacles for widespread adoption. Numerous studies have demonstrated that RFID systems can interfere with medical devices [1-8] and previous ad hoc testing by FDA [8] demonstrated the need for a standardized test method, as it is impractical and time consuming to test for EMC between a medical device and individual RFID systems. RFID signal output, field strength, frequency, and separation distance are all factors that can contribute to the likelihood of electromagnetic interference (EMI). IEC 60601-1-2:2007 (the EMC test standard for non-implantable medical devices) has no immunity requirements at 125 kHz nor a radiated immunity requirement at 13.56 MHz. Additionally, the radiated immunity requirements at 915 MHz and 2.4 GHz do not represent the potential field strengths allowed by the FCC. As such, medical devices tested to IEC 60601-1-2 can still be susceptible to RFID emissions as shown by Seidman et al. [1,8].

Because there are currently no standards that specify tests for medical device immunity to RFID emissions, medical device manufacturers typically demonstrate immunity through ad hoc testing, which has been shown to be both time consuming and labor intensive [8]. Previously it has been shown that by utilizing an RFID signal simulator it might be possible to test for a variety of RFID signals and field strengths, without the need for actual RFID systems [9]. Simulators described in detail in [10] are intended to provide an alternative means of testing for RFID emissions that is both faster and more reproducible. This type of testing would also provide medical device manufacturers with a better understanding of the RFID systems that could affect their devices, and this information can be used to improve device design.

The research described in this paper was performed to determine (1) simulator testing feasibility and adequacy and (2) if a broad range of input signals is necessary or if the test library can be simplified. Evaluation of the feasibility and adequacy of this simulator and the test library is important to support their incorporation into applicable medical device EMC standards.

## Methods

**Figure 1:**
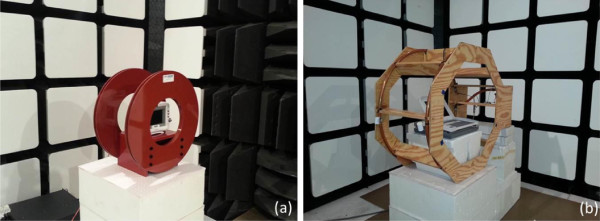
**Magnetic field exposure systems.** Photos of 125 kHz **(a)** and 13.56 MHz **(b)** exposure systems.

**Figure 2:**
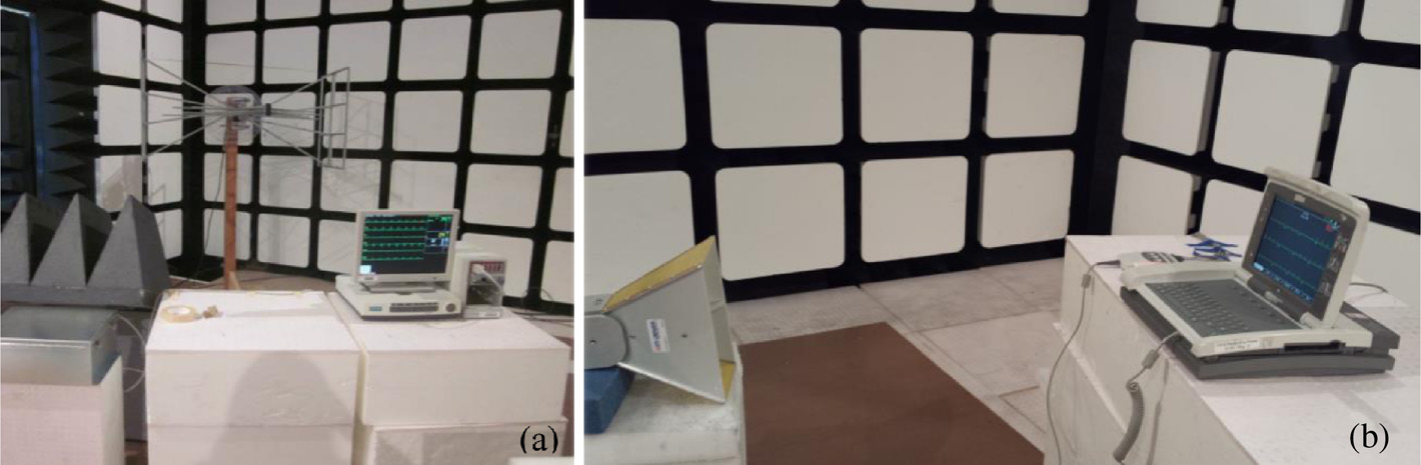
**Electric field exposure systems.** Photos of 915 MHz **(a)** and 2.4 GHz **(b)** exposure systems.

As discussed in Seidman et al. [10], separate simulators were developed to cover four distinct RFID frequency bands: Low frequency (LF): 125 kHz; High frequency (HF): 13.56 MHz; Ultra high frequency (UHF): 915 MHz; and 2.4 GHz. At 125 kHz and 13.56 MHz, Helmholtz Coils were designed to produce the radiated magnetic field strength (see Figure 1). At 915 MHz and 2.4 GHz, the exposure setup was similar to that described by IEC 61000-4-3, with an adjusted input signal and field strength according to the RFID test library (see Figure 2). The input signals are intended to emulate the applicable RFID standards with the various RFID reader settings adjusted to create a signal with the maximum and minimum occupied bandwidth. A summary of these signals, named the RFID Test Library is presented in Table 1.

**Table 1:** The RFID Test library

**Frequency**	**Occupied bandwidth**	**Standard**	**Program settings**	**Maximum field strength**
125 kHz (LF)	Max	ISO 14223 Type A	Modulation Depth 100%	65 A/m
			ETU: 3 usec	
	Min	ISO 14223 Type A	Modulation Depth 90%	65 A/m
			ETU: 12 u sec	
13.56 MHz (HF)	Max	ISO 14443 Type A	Pulse Width: 3 usec	12 A/m
			Transition Edge: 1 nsec	
	Min	ISO 14443 Type A	Pulse Width: 2 usec	12 A/m
			Transition Edge: 2.98 usec	
	Max	ISO 14443 Type B	Modulation Depth: 25%	12 A/m
			Transition Edge: 0.001 usec	
			SOF Low: 11 etu	
			SOF High: 2.5 etu	
			EOF Low: 10.5 etu	
	Min	ISO 14443 Type B	Modulation Depth: 15%	12 A/m
			Transition Edge: 1.17 usec	
			SOF Low: 10 etu	
			SOF High: 2 etu	
			EOF Low: 10 etu	
	Max	ISO 15693	Modulation Depth: 100%	12 A/m
			Pulse Width: 9.44 usec	
			Transition Edge: 1 nsec	
	Min	ISO 15693	Modulation Depth: 10%	12 A/m
			Pulse Width: 7.5 usec	
			Transition Edge: 800 nsec	
915 MHz (UHF)	Max	ISO 18000-6C	Modulation: DSB-ASK	54 V/m
			Tari: 6.25 us	
			Pulse Width: 3.281 usec	
			Modulation Depth: 100%	
	Max	ISO 18000-6C	Modulation: DSB-ASK	54 V/m
			Tari: 25 us	
			Pulse Width: 0.265 Tari	
			Modulation Depth: 80%	
	Min	ISO 18000-6C	Modulation: PR-ASK	54 V/m
			Tari: 6.25 usec	
			Pulse Width: 1.656 usec	
	Min	ISO 18000-6C	Modulation: PR-ASK	54 V/m
			Tari: 25 usec	
			Pulse Width: 0.525 Tari	
2.45 GHz	Max	ISO 18000–4 Mode 1	Modulation Depth: 100%	54 V/m
			Transition Edge: 1 nsec	
			Tari: 25 usec	
	Min	ISO 18000–4 Mode 2	Modulation Depth: 90%	54 V/m
			Transition Edge: 400 nsec	
			Tari: 33 usec	

The RFID Test Library is a collection of test signals and field strengths that are representative for most commercially available RFID readers (RMS).

An infusion pump and three different patient monitors were tested against the RFID Test Library. To begin, each medical device was placed on a non-conductive surface and configured for normal operation. Normal operation for the infusion pump was specified as delivering saline at its minimum flow rate of 100 ml/hr. Normal operation for the patient monitors was correct detection and analysis of signals from a patient simulator (Dynatech Nevada Inc, Model 214A) set to output a normal sinus rhythm at 80 beats per minute to the acquisition module of the monitor. The patient simulator was placed in a shielded box to exclude any EMI that might have occurred with the simulator itself. We began testing the patient monitor with the acquisition module exposed as it would be configured in clinical use. However, the acquisition modules were extremely susceptible to EMI, and as the objective of this testing was to determine the feasibility of the test protocol and appropriateness of the RFID Test Library; we decided to gather additional data with the acquisition module shielded from exposure.

After normal operation of the device was confirmed, each device was exposed to an RFID test signal at maximum field strength from the RFID test library. Each device was exposed to four orientations for each RFID test signal: front, back, left, and right. Depending on the implementation and geometry of the device it could be helpful to expose the top and bottom orientation as well. Magnetic field testing in a Helmholtz coil is somewhat more efficient than electric field radiated testing in a chamber because the parallel (or perpendicular) orientation exposes both the front and back (or left and right) sides simultaneously. All medical devices were visually and audibly monitored during exposure with a surveillance video camera. Any observable changes in device operation were recorded for all tests and classified based on the severity of the interference. If EMI was observed at the maximum field strength, we performed an additional test to discover the threshold at which EMI began. During threshold testing, the field strength was increased in increments of approximately 10% from 1 A/m to 12 A/m, until the first instance of EMI was observed. After EMI occurred, the field strength was lowered and the device was verified to return to normal operation. Next the field was returned to the level that caused the EMI and the threshold was verified. The dwell time for each exposure was 15 seconds.

## Results

Observed EMI ranged from device mode changes to screen errors. Device mode changes and screen errors that made display information unreadable were considered probably clinically significant (Class I). Small display changes where the display information was still identifiable were considered probably not clinically significant (Class II).

We observed EMI (Class I or II) during 24% (75 of 312) of our maximum field strength experiments with 76% (57 of 75) being classified as Class I. EMI was observed during 25% (6 of 24) of maximum field strength tests at 125 kHz; during 100% (48 of 48) of maximum field strength tests at 13.56 MHz; during 12% (19 of 160) of maximum field strength tests at 915 MHz; and during 3% (2 of 80) of maximum field strength tests at 2.4 GHz. The numbers above are inclusive of all tests, including experiments that were originally performed with the patient acquisition module exposed; for the remainder of this section we will ignore those experiments to minimize variables and concentrate purely on the appropriateness of the RFID Test Library.

During maximum field strength exposure to 13.56 MHz test signals, we observed EMI in 100% (48 of 48) of the tests. Class I EMI was observed for all three patient monitors and Class II EMI was observed for the infusion pump. No EMI was observed during exposure to 125 kHz (0 of 16), 915 MHz (0 of 128) and 2.4 GHz (0 of 64) RFID test signals.

At the maximum field strength it was not possible to determine any effect of different RFID test signals within each frequency band because every 13.56 MHz RFID test signal caused EMI. Thus for these cases where EMI was observed, we also found the field strength threshold where EMI began. We could then use these threshold values to compare the effects of different RFID tests signals within each frequency band. There were six 13.56 MHz RFID test signals (three different standards each tested at both maximum and minimum bandwidth). Results for these tests are presented in Table 2 below:

**Table 2:** Threshold EMI values at 13.56 MHz for parallel and perpendicular orientations for all devices

**Device #**	**RFID Standard protocol**	**Threshold H-Field strength (A/m RMS)**	
		**Parallel**	**Perpendicular**
1	ISO 14443 Type A Max	4.5	4.5
	ISO 14443 Type A Min	4.5	6.5
	ISO 14443 Type B Max	4.5	4
	ISO 14443 Type B Min	4	4
	ISO 15693 Max	4	3.5
	ISO 15693 Min	4.5	3.5
2	ISO 14443 Type A Max	10.5	12
	ISO 14443 Type A Min	10.5	12
	ISO 14443 Type B Max	10.5	9.5
	ISO 14443 Type B Min	10.5	12
	ISO 15693 Max	10.5	11.5
	ISO 15693 Min	10.5	11.5
3	ISO 14443 Type A Max	12	12
	ISO 14443 Type A Min	12	12
	ISO 14443 Type B Max	12	12
	ISO 14443 Type B Min	12	12
	ISO 15693 Max	12	12
	ISO 15693 Min	12	12
4	ISO 14443 Type A Max	12	12
	ISO 14443 Type A Min	12	12
	ISO 14443 Type B Max	12	12
	ISO 14443 Type B Min	12	12
	ISO 15693 Max	12	12
	ISO 15693 Min	12	12

## Discussion

This study aimed to determine (1) simulator testing feasibility and adequacy and (2) if all proposed input signals are necessary or if the test library can be simplified. Test feasibility and adequacy was verified by demonstrating that the simulator can be used to determine medical device-RFID EMC. Little variation was seen comparing the different RFID protocols and the maximum versus minimum bandwidth. The average difference comparing threshold field strengths between different standards was only 0.25 A/m and the average difference comparing the maximum versus the minimum bandwidth was only 0.23 A/m. Additionally, identical EMI was observed for all patient monitors tested across the six protocols. While only four medical devices were tested, this data collected tends to suggest that, while signal modulation has an effect, the greater influence is the overall field strength. This would support minimizing the RFID Test Library to one signal per frequency. However, threshold testing was only performed at 13.56 MHz and more testing with more medical devices is needed to verify simplification of the RFID Test Library. Since little variation was seen between the different RFID protocols future tests should consider whether the specified modulation defined in IEC 60601-1-2:2007 is adequate with the appropriate higher test levels.

Varying EMC performance was seen between devices. Device 1 exhibited EMI at an average field strength of 4.3 A/m, while all of the other medical devices exhibited EMI at an average field strength between 11 A/m and 12 A/m. While our results confirm the potential of RFID EMI to medical devices, we do not recommend using this data for clinical decisions. First, there were only four devices tested in this study and they do not represent a broad enough selection of medical device types or manufacturers. Secondly, the devices were overexposed for a portion of the testing performed. A maximum test level of 12 A/m was chosen at 13.56 MHz for all three different standards. However, while ISO 18000–3 Mode 1 specifies a maximum field strength of 12 A/m, ISO/IEC 14443 and ISO/IEC 15693 specify 7.5 A/m and 5 A/m, respectively (over the manufacturer’s specified volume). Similarly the 54 V/m test level at 2.4 GHz is a theoretical maximum at 20 cm. In our experience the field values from 2.4 GHz readers are less than 2 V/m at this distance. Finally, during threshold testing the patient monitor acquisition modules were shielded from interference. Many medical devices sense vital signs from the body and a modulated RF signal can be misinterpreted as one coming from a patient by that unit if it is exposed. In clinical use, the acquisition module would be exposed, resulting in EMI at lower field strengths than those reported here.

A side by side comparison of our simulator data to actual RFID readers would be helpful to demonstrate that our test accurately portrays exposure from actual RFID readers. However, there are some practical reasons why these tests were not performed. Primarily this was not performed because testing to a maximum field in a volume or over an area as described in our test method is generally understood to be a more rigorous exposure than testing with an actual RFID reader, where the maximum field will be localized and decrease with distance. This means that passing our test is more difficult than passing a test with actual RFID readers. Additionally, we were not able to reproduce all of the field levels with actual RFID readers, as explained in the prior paragraph. We did do some testing using actual RFID readers as the signal input, but with our same exposure system. As expected, this data was consistent with that found from using the signals from the RFID Test Library.

## Conclusions

Medical devices have been shown to be susceptible to EMI from RFID and current medical device EMC standards do not adequately test electromagnetic immunity of medical devices to RFID systems. This study has demonstrated the feasibility and adequacy of using simulators and the RFID Test Library to test medical devices for EMC with RFID. Using simulators, we have shown that testing can be performed much faster than in ad hoc testing with individual RFID systems. The results from our tests demonstrate that, while signal modulation has an effect on medical devices, the greater influence is the overall field strength. Tests are also more repeatable than in ad hoc testing because the hardware configuration remains unchanged from test to test and results are not affected by small changes in antenna orientation.

Medical device manufacturers could use these simulators to test if their devices are susceptible to RFID emissions. This test can be used to assist the medical device community to identify and resolve potential EMI that could be caused by RFID systems and provide assurance to hospitals that RFID technology will not adversely affect their medical devices. It has also been shown to be suitable for incorporation into applicable consensus standards.

## Abbreviations

RFID: Radio frequency identification; EMC: Electromagnetic compatibility; EMI: Electromagnetic interference; LF: Low frequency; HF: High frequency; UHF: Ultra high frequency.

## Competing interests

The authors declare that they have no competing interests.

## Authors’ contributions

All authors have made substantial contributions to the conception and design, acquisition, analysis and interpretation of data. All authors were involved in drafting the manuscript. All authors read and approved the final manuscript.
